# NAT10‐Mediated ac4C Modification of circANKRD12 Reprograms the Tumor Microenvironment

**DOI:** 10.1002/advs.75797

**Published:** 2026-05-22

**Authors:** Jiale Zhang, Hui Shi, Chen Wang, Zihao Liu, Lianxin Zhou, Xinyu Lv, Mengjie Guo, Ye Yang, Chunyan Gu

**Affiliations:** ^1^ Nanjing Hospital of Chinese Medicine Affiliated Hospital of Nanjing University of Chinese Medicine Nanjing China; ^2^ School of Medicine Nanjing University of Chinese Medicine Nanjing China

**Keywords:** circANKRD12, desloratadine, multiple myeloma, N4‐acetylcytidine, N‐acetyltransferase 10

## Abstract

Developing anticancer strategies that simultaneously target both tumor proliferation and the immunosuppressive microenvironment remains a major challenge. However, the role of chemical modifications in circular RNAs (circRNAs) in this process remains poorly understood. In this study, we identified circANKRD12 as a key substrate for N^4^‐acetylcytidine (ac4C) modification, catalyzed by N‐acetyltransferase 10 (NAT10) in multiple myeloma (MM). This ac4C modification promotes the translation of circANKRD12 into a novel 354‐amino acid protein (circANKRD12_354aa). Functionally, circANKRD12_354aa interacts with histone deacetylase 2 (HDAC2) to stabilize the oncoprotein c‐Myc, thereby driving MM cell proliferation. Moreover, circANKRD12 could be transferred from MM cells to natural killer (NK) cells, where it similarly suppressed NK cell cytotoxicity via the HDAC2/c‐Myc axis, facilitating immune evasion. Clinically, circANKRD12 was upregulated in MM patients and correlated with poorer prognosis. Through high‐throughput screening, we further identified the clinical antihistamine desloratadine as a direct binder of circANKRD12_354aa. Targeting the circANKRD12/HDAC2/c‐Myc axis with desloratadine effectively suppresses MM growth and restores NK cell‐mediated antitumor immunity in vivo. Our study reveals that NAT10‐mediated ac4C modification of circANKRD12 plays a central role in coordinating tumor proliferation and immune dysfunction, establishing circANKRD12_354aa as a promising therapeutic target for restoring antitumor immunity in MM.

## Graphical abstract

NAT10‐dependent acetylation of circANKRD12 drives translation of the circANKRD12_354aa protein, which binds HDAC2 to stabilize c‐Myc via deubiquitination, promoting multiple myeloma (MM) cell proliferation. Concurrently, the circANKRD12‐HDAC2 axis suppresses H3ac‐mediated transcription of IFN‐γ, TNF‐α, and GZMB in NK cells, leading to NK cell dysfunction. Disrupting this circuit restores NK cell activity and inhibits MM progression.

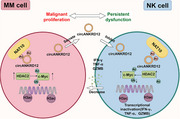

**Abbreviations**
ac4C: N4‐acetylcytidine
acRIP‐seq: acetylated RNA immunoprecipitation sequencing
ANKRD12: ankyrin repeat domain 12
BMD: bone mineral density
BV/TV: bone volume/trabeculae volume
CCK‐8: cell counting kit 8
CDK4: cyclin‐dependent kinase 4
ChIP‐seq: chromatin immunoprecipitation sequencing
c‐Myc: cellular myelocytomatosis oncogene
Co‐IP: co‐immunoprecipitation
EFS: event‐free survival
ELISA: enzyme linked immunosorbent assay
GZMB: granzyme B
H3: histone 3
HDAC2: histone deacetylase 2
IF: immunofluorescence
IFN‐γ: interferon‐γ
KRAS: kirsten rat sarcoma viral oncogene homolog
LDH: lactate dehydrogenase
MM: multiple myeloma
MST: microscale thermophoresis
NAT10: N‐acetyltransferase 10
NC: negative control
NK: natural killer
RT‐qPCR: reverse transcription quantitative polymerase chain reaction
siRNA: small interfering RNA
TME: tumor microenvironment
TNF‐α: tumor necrosis factor‐α
WB: western blot
WT: wild type

## 1 Introduction

The combination of immunotherapy and chemotherapy has yielded impressive anti‐tumor responses in many patients [1, 2, 3]. However, the clinical efficacy of such regimens is often limited by intrinsic or acquired resistance, representing a major therapeutic challenge. This limitation underscores a fundamental biological gap: the disconnect between tumor‐intrinsic proliferative pathways and a suppressed or dysfunctional anti‐tumor immune response within the tumor microenvironment (TME). Therefore, identifying molecular nodes that co‐regulate both malignant proliferation and immune evasion is essential for developing next‐generation strategies that can simultaneously target tumor cells and restore effective anti‐tumor immunity.

Circular RNAs (circRNAs) are a widespread class of endogenous non‐coding RNAs that are generated by back‐splicing of pre‐mRNA transcripts. They have been identified as important regulators in cancer biology [4], playing a role in processes such as cell proliferation, metastasis, and drug resistance. Due to their inherent stability and presence in exosomes, they also play a crucial role in intercellular communication within the tumor microenvironment (TME). Our previous research has shown that specific circRNAs are critical in the progression of multiple myeloma (MM), drug resistance, and remodeling of the bone marrow niche through their transfer between myeloma cells and stromal components, such as osteoclasts [5, 6, 7, 8]. Additionally, there is growing evidence that circRNAs also play a role in regulating anti‐tumor immunity by modulating the function of various immune cells [9, 10]. These findings suggest that circRNAs may serve as important molecular links connecting the malignant behavior of MM cells to the dysfunctional state of the local immune landscape. Therefore, it is crucial to systematically identify circRNAs that co‐regulate MM cell proliferation and anti‐MM immune dysfunction, making it a strategic research priority.

The functional diversity of circRNAs is tightly regulated by post‐transcriptional modifications. N‐acetyltransferase 10 (NAT10), the sole known writer for the RNA modification N4‐acetylcytidine (ac4C), enhances the translation efficiency and stability of its target RNAs [11]. Accumulating evidence links NAT10 to tumor progression [12, 13]. Our prior study demonstrated that NAT10 acetylates *CEP170* mRNA to promote MM progression [14]. Although ac4C modification was initially characterized in rRNAs, tRNAs, and mRNAs [15], its presence and functional impact on circRNAs remain entirely unexplored. This knowledge gap prompted our hypothesis: could NAT10‐mediated ac4C modification serve as a novel regulatory mechanism controlling the biogenesis, stability, or function of oncogenic circRNAs in MM?

In this study, we have identified circANKRD12 as a crucial substrate for NAT10‐mediated ac4C modification in MM. Our findings demonstrate that this ac4C modification plays a critical role in regulating circANKRD12 function, promoting MM cell proliferation while simultaneously suppressing the cytotoxicity of natural killer (NK) cells. This dual effect reveals a previously unrecognized signaling pathway through which a single circRNA coordinates both tumor growth and immune evasion within the microenvironment.

## 2 Materials and Methods

### 2.1 Cell Culture

Human MM cell lines ARP1 and KMS28PE were generously provided by Prof. Siegfried Janz (Medical College of Wisconsin, Milwaukee, WI, USA). The 5TMM3VT and HEK‐293 cell lines were gifts from Prof. Wen Zhou (Central South University, China). ARP1, KMS28PE, and 5TMM3VT cells were maintained in RPMI‐1640 medium (01‐100‐1ACS, Biological Industries), while HEK‐293 cells were cultured in Dulbecco's modified Eagle's medium (DMEM, 01‐052‐1ACS, Biological Industries). The human NK cell line NK92/MI was grown in ready‐to‐use α‐MEM complete medium (Cell Cook, Cat. No. CC1625S, Guangzhou, China). All media were supplemented with 10% fetal bovine serum (FBS, 04‐001‐1ACS, Biological Industries). Cells were incubated at 37°C in a humidified atmosphere with 5% CO_2_. Cell lines were also regularly evaluated for mycoplasma contamination using the Mycoplasma PCR Detection Kit (Beyotime, Cat. No. C0301S, Shanghai, China).

### 2.2 Plasmid Construction and Lentiviral Transduction

The coding sequence of Homo circANKRD12 (NM‐001204056.1) was synthesized by TranSheepBio and cloned into the pLC5 lentiviral vector with an HA tag. Lentiviral particles were produced by co‐transfecting HEK‐293 cells with the pLC5‐circANKRD12 construct and packaging plasmids (PSPAX.2 and PMD2.G) using Lipofectamine transfection reagent. After 48 h, the viral supernatant was collected, concentrated, and stored at –80°C. MM cells were transduced with the lentivirus to generate circANKRD12‐overexpressing (circANKRD12‐OE) cells. Transduced cells were selected with puromycin, and overexpression efficiency was confirmed by Western blot and RT‐qPCR.

### 2.3 Small Interfering RNA (siRNA) Transfection

The cells were transfected with siRNA using an electroporation system (BTX). To begin, the cells were suspended in BTX Cytoporation Media T4 (47‐0003) at a density of 1 × 10^6^/mL. Next, the cells were mixed with siRNA to a final concentration of 100 nm and transferred to an electroporation cuvette. The electroporation process was carried out using the following parameters: square wave, 960 V, 0.1 ms pulse duration, 2 pulses, 1.0 s interval, and 4 mm electrode gap. All siRNAs were synthesized by Shanghai GenePharma, and their sequences are listed in Table S1.

### 2.4 Real‐Time Quantitative PCR

Total RNA was extracted using the TRIeasy reagent. cDNA was synthesized using a reverse transcription kit according to the manufacturer's protocol. RT‐qPCR was performed using SYBR Green master mix. Primer sequences are provided in Table S2.

### 2.5 Biosequencing

acRIP‐seq was conducted as previously described. Data analysis was performed by Guangzhou Epibiotek Co., Ltd. (Guangzhou, China). ChIP‐seq sequencing was carried out by Novogene (Beijing, China). The raw sequence data for this study have been deposited in the Genome Sequence Archive (Genomics, Proteomics & Bioinformatics 2025) in the National Genomics Data Center (Nucleic Acids Res 2025), China National Center for Bioinformation / Beijing Institute of Genomics, Chinese Academy of Sciences (GSA: CRA037118), which are publicly accessible at https://ngdc.cncb.ac.cn/gsa.

### 2.6 Western Blot and Co‐Immunoprecipitation

Western blot (WB) was performed using standard protocols. Co‐immunoprecipitation (Co‐IP) was conducted using the Pierce Direct Magnetic IP/Co‐IP kit (Thermo Scientific) according to the manufacturer's instructions. Antibody information is listed in Table S3.

### 2.7 Immunofluorescence (IF) and Confocal Microscopy

Cells were fixed with 4% paraformaldehyde, permeabilized with 0.1% Triton X‐100 in PBS, and treated with 50 mm NH_4_Cl for 5 min to quench autofluorescence. After blocking with 1% BSA, cells were incubated with primary antibodies overnight at 4°C, then with fluorophore‐conjugated secondary antibodies. Images were acquired using a Leica TCS SP8 confocal microscope.

### 2.8 Giemsa Staining

Giemsa staining was performed using a rapid staining kit (BBI Life Sciences) according to the manufacturer's instructions.

### 2.9 Mass Spectrometry

Proteins were separated by SDS‐PAGE, and target gel bands were excised and digested with sequencing‐grade trypsin (Promega). The resulting peptides were analyzed using a QExactive mass spectrometer (Thermo Fisher Scientific). Spectra were matched against the NCBI nonredundant protein database. The MS data reported in this study are stored on the iProX platform (PXD072986) that are publicly accessible at https://www.iprox.cn.

### 2.10 MM Xenograft Model

ARP1 wild‐type, circANKRD12‐OE, and circANKRD12‐MUT cells (1 × 10^6^) were subcutaneously injected into the left and right flanks of 6–8‐week‐old NOD‐SCID mice, respectively. Tumor dimensions were measured daily. When the tumor diameter reached 15 mm, the mice were euthanized, and the tumors were excised, weighed, and photographed. All animal procedures were approved by the Institutional Ethics Committee of Nanjing University of Chinese Medicine (Ethics No. 202406A031) and were conducted in accordance with national guidelines for laboratory animal care.

### 2.11 5TMM3VT Mouse Model

C57BL/KaLwRij mice (Harlan Laboratories) were injected via the tail vein with 1 × 10^6^ 5TMM3WT empty vector (EV) or circANKRD12‐OE cells (*n* = 6 per group) [14]. Desloratadine was administered at a dose of 10 mg/kg body weight via intraperitoneal injection every other day for a period of 4 weeks, with the control group receiving only the vehicle (5% DMSO in sterile PBS, 100 µL per mouse).

### 2.12 Pristane‐Induced Model

Balb/c mice (4–5 weeks old, sex‐balanced) were administered AAV co‐expressing GFP and HA tags via the tail vein, followed by three intraperitoneal injections of pristane at 2 month intervals to induce plasma cell tumors [16].

### 2.13 Enzyme‐Linked Immunosorbent Assay (ELISA)

After culturing for 48 h, the supernatants from NK92/MI cells were collected and then centrifuged at 1200 rpm for 1 min. The levels of TNF‐α and IFN‐γ were then measured using an ELISA kit (Yi Feixue, China) following the manufacturer's instructions.

### 2.14 LDH Cytotoxicity Assay

Cytotoxicity was assessed by measuring LDH release in treated NK92/MI cell supernatants using a commercial kit (Beyotime, China).

### 2.15 Chromatin Immunoprecipitation (ChIP)

ChIP was performed using a kit from Cell Signaling Technology. First, cells were crosslinked with 1% formaldehyde, then lysed and sonicated. Next, the lysates were incubated with specific antibodies and protein A/G magnetic beads. The precipitated DNA was then eluted, reverse‐crosslinked, and purified. The level of enrichment was measured using RT‐qPCR and normalized to IgG controls. The primer sequences used are provided in Table S4.

### 2.16 Construction of Mutant Plasmids

For circANKRD12‐MUT, based on the ac4C modification site predicted by the PACES tool (residues 112–126 of circANKRD12), we mutated the predicted acetylated cytidine to adenosine, which abrogates acetylation and subsequent translation of the circANKRD12_354aa protein. The mutation was introduced into the pLC5‐circANKRD12 overexpression plasmid by site‐directed mutagenesis, with the exact nucleotide substitutions being C112G and C115G. For c‐Myc‐MUT, we generated an acetylation‐deficient mutant by mutating the known HDAC2‐mediated deacetylation sites. Specifically, lysine (K) residues at positions 148 and 157 within the c‐Myc coding sequence were substituted with arginine (R), thereby preventing HDAC2‐dependent deacetylation and subsequent stabilization of c‐Myc. This mutant was cloned into the pcDNA3.1+ vector. All constructs were verified by Sanger sequencing. Detailed information on the mutagenesis primers and the resulting sequences is provided in Table S5.

### 2.17 Mouse Primary NK Cell Isolation

Peripheral blood was collected from mice via orbital bleeding and centrifuged at 300 g for 10 min. Erythrocytes were lysed using RBC lysis buffer, and NK cells were isolated using CD49b MicroBeads (Miltenyi Biotec, Germany) according to the manufacturer's protocol.

### 2.18 Prediction of ac4C Sites in circANKRD12

The PACES online tool (http://rnanut.net/paces/) was used to predict ac4C modification sites within circANKRD12.

### 2.19 Statistical Analysis

Statistical analyses were performed using GraphPad Prism 9.5.1. Data are presented as mean ± SD. Comparisons between two groups were performed using two‐tailed unpaired Student's *t*‐tests. For comparisons involving multiple groups, two‐way ANOVA followed by Sidak's or Tukey's post hoc test was used. Survival differences were assessed by the log‐rank test. Statistical significance is denoted as ^*^ *p* < 0.05, ^**^ *p* < 0.01, and _***_ *p* < 0.001; n.s., not significant.

## 3 Results

### 3.1 NAT10‐Mediated ac4C Modification Promotes circANKRD12_354aa Translation

**FIGURE 1:**
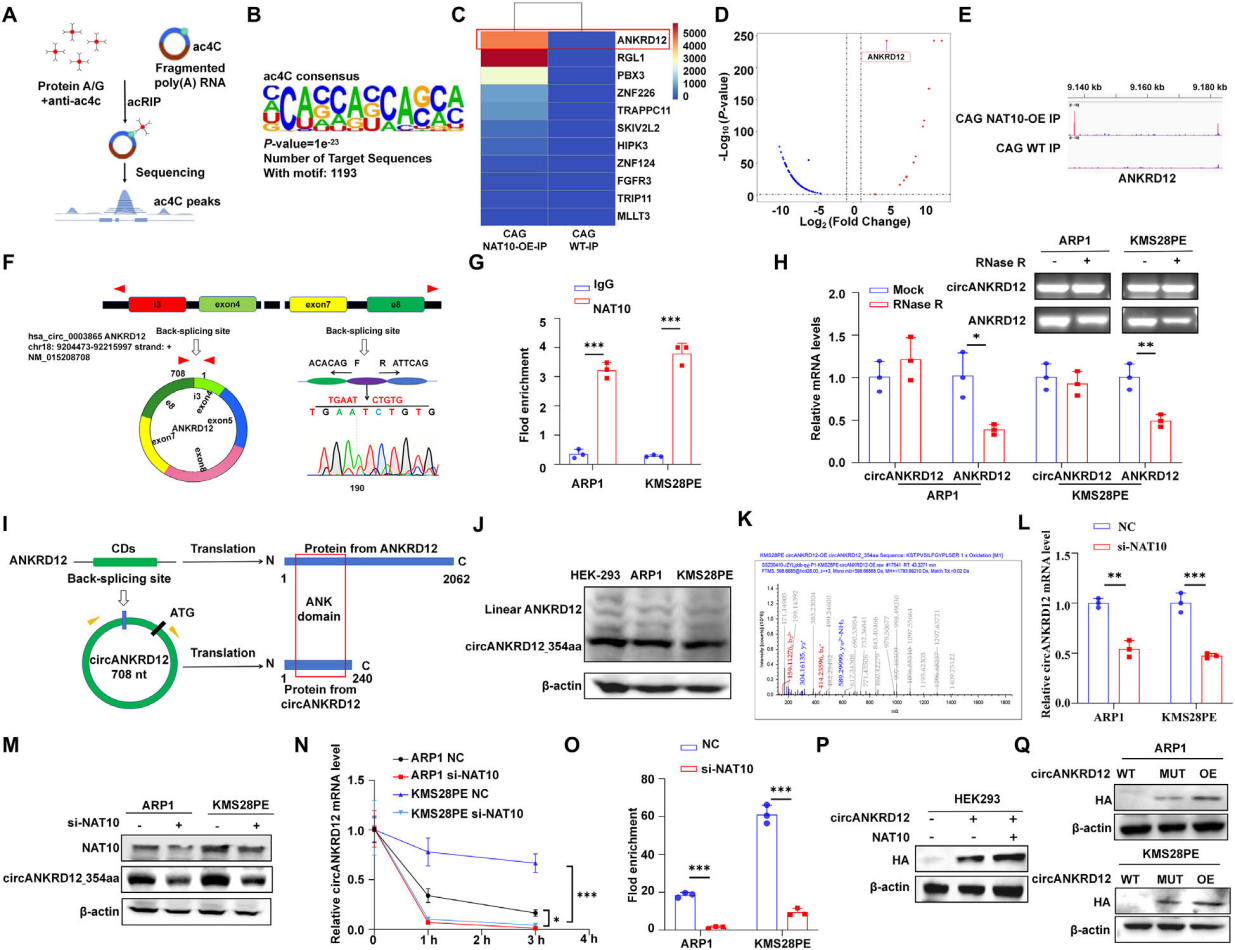
NAT10‐mediated ac4C modification promotes circANKRD12_354aa translation. (A) Schematic of the acRIP‐seq workflow. (B) Representative CXX motif identified within ac4C enrichment peaks. (C) Heatmap showing genes with co‐upregulated acetylation in CAG NAT10‐OE cells. (D) Volcano plot of RNAs with increased (red) or decreased (blue) acetylation levels upon NAT10 overexpression. (E) ac4C enrichment peaks at the ANKRD12 locus in CAG WT and NAT10‐OE IP samples (*n* = 3). (F) Genomic annotation of ANKRD12, predicted RNA splicing isoforms, and Sanger sequencing validation of circular exon 4–7 (circANKRD12). (G) RIP‐qPCR confirming NAT10 binding to circANKRD12 in MM cells (*n* = 6). (H) RNA levels of circANKRD12 and linear ANKRD12 with or without RNase R treatment were assessed by PCR and RT‐qPCR (*n* = 6). (I) Schematic of linear and circular ANKRD12 coding regions. (J) Basal expression level of the protein encoded by circANKRD12. (K) Mass spectrometry validation of circANKRD12 protein‐coding potential (*n* = 3). (L) The expression levels of circANKRD12 in ARP1 and KMS28PE cells were detected by RT‐qPCR after si‐NAT10 treatment (*n* = 6). (M) CircANKRD12_354aa protein expression in ARP1 and KMS28PE cells was detected by WB after si‐NAT10 treatment. (N) Half‐life of circANKRD12 in ARP1 and KMS28PE cells was detected by RT‐qPCR after si‐NAT10 treatment (*n* = 6). (O) Acetylation level of circANKRD12 in ARP1 and KMS28PE cells was detected by acRIP‐qPCR after si‐NAT10 treatment (*n* = 6). (P) WB analysis of protein expression in HEK‐293 cells co‐transfected with NAT10 and circANKRD12 plasmids. (Q) WB analysis in ARP1 and KMS28PE cells transfected with an acetylation‐site mutant of circANKRD12. Comparisons among three or more groups were performed by using one‐way analysis of variance (ANOVA) followed by Dunnettʹs post hoc tests. All data are displayed as mean ± SD; ^*^ *p* < 0.05, ^**^ *p* < 0.01, ^***^ *p* < 0.001.

To identify circRNAs modified by ac4C, we performed acRIP‐seq on previously constructed NAT10‐overexpressing (NAT10‐OE) and wild‐type (WT) cells (Figure 1A). A characteristic CXX motif within the ac4C peaks confirmed the quality of the acRIP‐seq data (Figure 1B). We then screened for RNAs with elevated acetylation levels on the circbank database [17] (http://www.circbank.cn), where *ANKRD12* exhibited higher acetylation levels while forming circular structures (Figure 1C). Potential ac4C modification sites (positions 112–126) on *ANKRD12* were predicted using the PACES tool (Figure S1A–C) [18]. A volcano plot illustrated RNAs with significantly altered acetylation levels upon NAT10 overexpression, with upregulated and downregulated candidates shown in red and blue, respectively (Figure 1D) [16]. Notably, ANKRD12 exhibited the most significantly enriched ac4C peaks compared to others induced by NAT10 overexpression (Figure 1E). We confirmed the endogenous formation of circANKRD12 from exons 4–7 of the ANKRD12 gene by Sanger sequencing (Figure 1F). The result of RIP‐qPCR examined the physical interaction between NAT10 and *circANKRD12* (Figure 1G). Then, circANKRD12 demonstrated resistance to RNase R digestion, whereas the linear ANKRD12 transcript was significantly degraded (Figure 1H).

Structural analysis suggested that circANKRD12 possesses protein‐coding potential (Figure 1I). Using an ANKRD12 antibody, we confirmed the presence of circANKRD12_354aa in HEK‐293, ARP1, and KMS28PE cells, which was recognized explicitly by the N‐terminus of the ANKRD12 antibody (Figure 1J). Mass spectrometry (MS) further validated specific peptide fragments derived from circANKRD12_354aa in the KMS28PE circANKRD12‐OE cell (Figure 1K). To investigate the regulatory relationship between NAT10 and circANKRD12, we performed RT‐qPCR in MM cells following NAT10 knockdown. Then, circANKRD12 expression was significantly downregulated in si‐NAT10‐treated groups compared to negative controls (NC) (Figure 1L). Western blot (WB) analysis showed decreased circANKRD12_354aa protein levels after si‐NAT10 transfection (Figure 1M). RT‐qPCR revealed a significantly reduced half‐life for circANKRD12 following NAT10 knockdown (Figure 1N). Then, acRIP‐qPCR assays showed that siRNA‐mediated NAT10 knockdown not only reduced circANKRD12 abundance but also decreased its ac4C modification level (Figure 1O), confirming that NAT10 binds and acetylates circANKRD12.

Given that ac4C modification promotes mRNA translation, we hypothesized that NAT10 enhances circANKRD12 translation. Co‐transfection of circANKRD12 and NAT10 plasmids into HEK‐293 cells increased circANKRD12_354aa protein levels (Figure 1P). To explore the functional impact of this modification in MM cells, we mutated the predicted acetylation site of circANKRD12. Compared to the circANKRD12‐overexpressing (circANKRD12‐OE) group, the circANKRD12‐MUT group showed lower protein expression (Figure 1Q). These data demonstrate that NAT10 mediates ac4C modification of circANKRD12 to promote the translation of circANKRD12_354aa.

### 3.2 CircANKRD12_354aa Binds to HDAC2 to Enhance the Expression of c‐Myc

**FIGURE 2:**
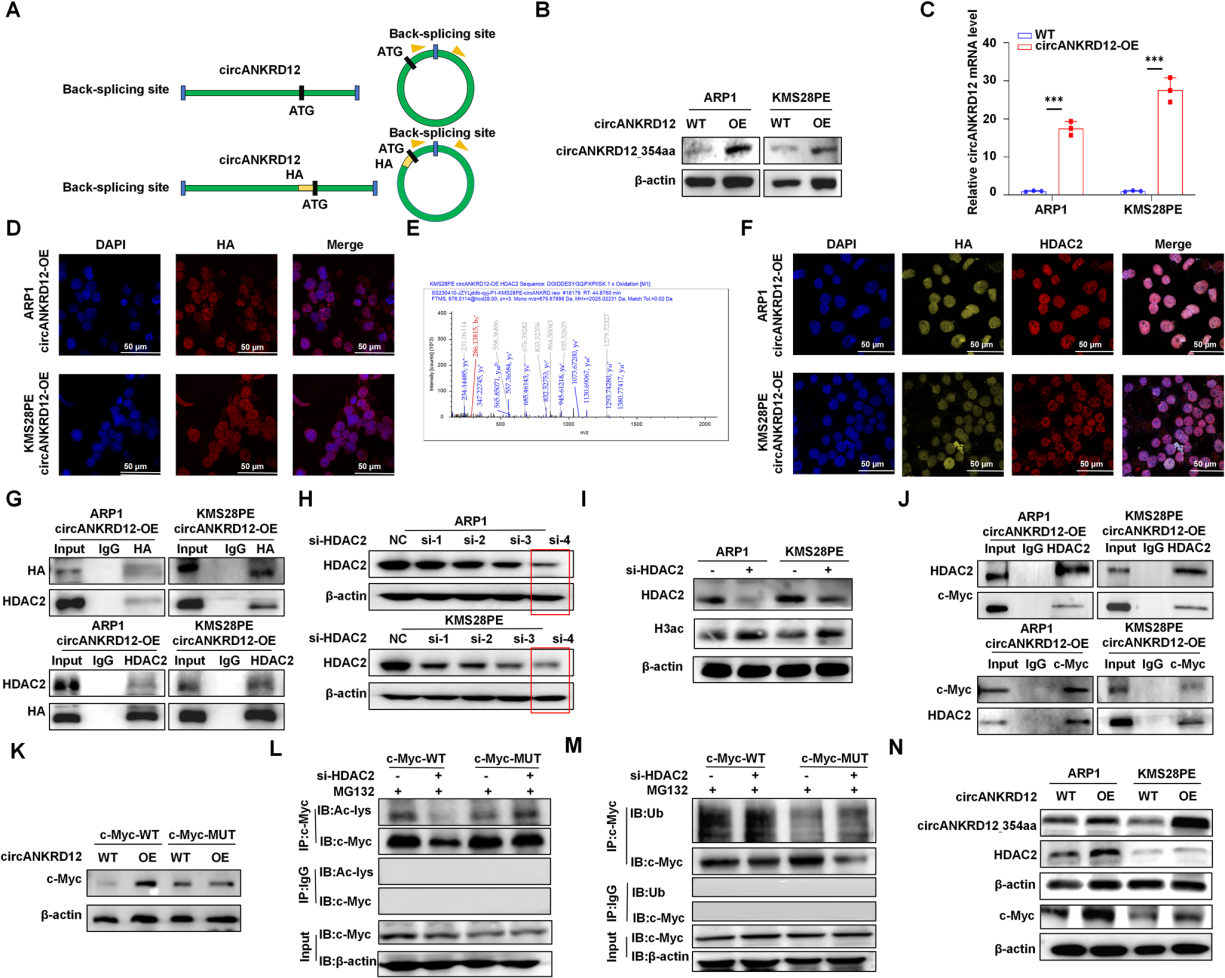
CircANKRD12_354aa binds to HDAC2 to enhance the expression of c‐Myc. (A) Schematic of the circANKRD12 overexpression plasmid construct. (B) WB confirmation of circANKRD12_354aa expression in circANKRD12‐OE cells. (C) RT‐qPCR validation of circANKRD12 expression in circANKRD12‐OE cells (*n* = 6). (D) IF analysis of circANKRD12_354aa subcellular localization in MM cells (*n* = 3). Scale bar: 50 µm. (E) MS identification of circANKRD12_354aa‐interacting proteins in KMS28PE circANKRD12‐OE cells (*n* = 3). (F) Confocal microscopy showing colocalization of circANKRD12_354aa with HDAC2 in MM cells (*n* = 3). Scale bar: 50 µm. (G) Co‐IP confirming the interaction between circANKRD12_354aa and HDAC2 in MM cells. (H) Decreased HDAC2 levels in ARP1 and KMS28PE cells transfected with siRNA‐4 (si‐4). (I) WB analysis of H3ac levels in MM cells after si‐HDAC2 treatment. (J) Co‐IP analysis of the interaction between HDAC2 and c‐Myc in MM cells. (K) WB detection of c‐Myc protein levels in MM cells. (L) Co‐IP assay examining the deacetylation of c‐Myc induced by si‐HDAC2 treatment. (M) Co‐IP analysis of si‐HDAC2 treatment‐mediated effects on c‐Myc ubiquitination. (N) Expression levels of HDAC2 and c‐Myc in circANKRD12‐OE MM cells. Comparisons among three or more groups were performed using one‐way analysis of variance (ANOVA) followed by Dunnett's post hoc tests. All data are displayed as mean ± SD; ^***^ *p* < 0.001.

To investigate the downstream mechanisms of circANKRD12, we constructed circANKRD12‐OE ARP1 and KMS28PE cells by inserting an HA tag into the circANKRD12 coding region. WB and RT‐qPCR analyses showed successful overexpression of circANKRD12 (Figure 2A–C). Subcellular localization via laser confocal microscopy revealed that circANKRD12_354aa primarily localizes to the nucleus (Figure 2D). To identify interacting proteins, we performed Co‐IP followed by MS in circANKRD12‐OE cells. HDAC2 was among the top proteins interacting with circANKRD12_354aa (Figure 2E). HDAC2, a key epigenetic modifier, deacetylates histones to regulate gene transcription [19]. Previous studies suggested that ANKRD12 functions as a transcriptional coactivator by recruiting HDACs [20]. The physical interaction between circANKRD12_354aa and HDAC2 was confirmed by Co‐IP and IF in circANKRD12‐OE cells (Figure 2F,G).

To knock down HDAC2 in MM cells, we designed siRNAs and verified knockdown efficiency using WB (Figure 2H). HDACs remove acetyl groups from histone H3 tails, resulting in tighter chromatin structure. HDAC2 knockdown significantly increased H3 acetylation (H3ac) levels (Figure 2I). Reports indicate that HDACs interact with c‐Myc, promoting its stability via deacetylation‐mediated inhibition of K48‐linked ubiquitination [21, 22]. The HDAC2/c‐Myc axis is critical for tumor proliferation and immune cell dysfunction [23]. Co‐IP confirmed the interaction between HDAC2 and c‐Myc (Figure 2J). To assess the effect of circANKRD12 and HDAC2 on c‐Myc, we mutated the binding sites of c‐Myc and co‐transfected c‐Myc‐WT or c‐Myc‐MUT plasmids with circANKRD12 into HEK‐293 cells. WB analysis showed that circANKRD12's ability to promote c‐Myc expression was weakened upon c‐Myc mutation (Figure 2K). Co‐IP showed that HDAC2 increased the acetylation level of c‐Myc‐WT but not c‐Myc‐MUT (Figure 2L). Similarly, the ubiquitination level of c‐Myc‐WT increased significantly after si‐HDAC2, whereas this effect was attenuated for c‐Myc‐MUT (Figure 2M). Furthermore, circANKRD12 overexpression significantly increased c‐Myc and HDAC2 protein levels (Figure 2N). These results demonstrate that circANKRD12 enhances the expression of HDAC2 and c‐Myc.

### 3.3 The circANKRD12/HDAC2 Axis Drives Divergent c‐Myc Transcriptional Programs to Promote MM Growth and Suppress NK Cell Function

Analysis of 10x Genomics high‐throughput sequencing data from the bone marrow of MM patients (GSE124310) and from our mouse model revealed NAT10 expression across various cell subsets (Figure S1A), with notably high expression in MM plasma cells (Figure S1B). Interestingly, compared with healthy controls, T cells, NK cells, and B cells exhibited distinct NAT10 expression patterns in the bone marrow of patients with MGUS and MM, with NK cells showing the highest expression (Figure S1C–E). Given the established role of HDAC2/c‐Myc signaling in tumor proliferation and NK cell cytotoxicity, and our finding that circANKRD12_354aa binds HDAC2/c‐Myc and upregulates c‐Myc expression, we further investigated the biological effects of this axis in MM and NK cells. Transcriptomic and c‐Myc ChIP‐seq analyses demonstrated that circANKRD12 differentially modulates c‐Myc transcriptional activity in these cell types.

**FIGURE 3:**
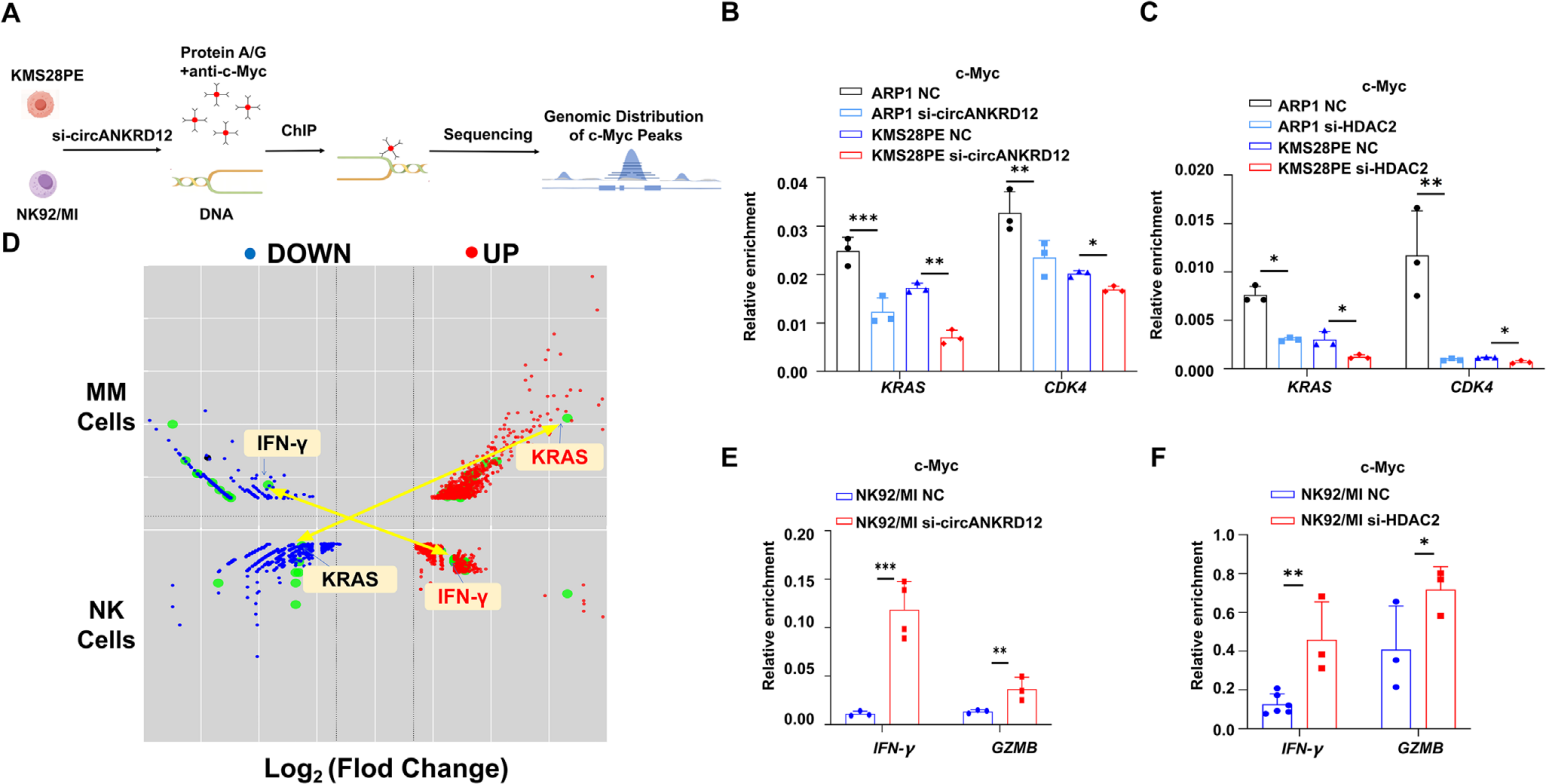
The circANKRD12/HDAC2 axis drives divergent c‐Myc transcriptional programs to promote MM growth and suppress NK cell function. (A) Schematic of the ChIP‐seq analysis of c‐Myc target genes in KMS28PE and NK92/MI cells following circANKRD12 knockdown. (B) ChIP‐qPCR analysis of c‐Myc enrichment on target gene promoters in ARP1 and KMS28PE cells after si‐circANKRD12 treatment (*n* = 6). (C) ChIP‐qPCR analysis of c‐Myc enrichment on target gene promoters in ARP1 and KMS28PE cells after si‐HDAC2 treatment (*n* = 6). (D) ChIP‐seq analysis of c‐Myc target genes in KMS28PE and NK92/MI cells following circANKRD12 knockdown (*n* = 3). (E) ChIP‐qPCR analysis of c‐Myc enrichment on target gene promoters in NK92/MI cells after si‐circANKRD12 treatment (*n* = 6). (F) ChIP‐qPCR analysis of c‐Myc enrichment on target gene promoters in NK92/MI cells after si‐HDAC2 treatment (*n* = 6). Comparisons among three or more groups were performed using one‐way analysis of variance (ANOVA) followed by Dunnett's post hoc tests. All data are displayed as mean ± SD; ^*^ *p* < 0.05, ^**^ *p* < 0.01, ^***^ *p* < 0.001.

In MM cells, circANKRD12 knockdown reduced c‐Myc binding to promoters of proliferation‐related genes (*KRAS, CDK4*) and increased its binding to promoters of NK cell effector genes (*IFN‐γ*, *GZMB*). Conversely, in NK cells, circANKRD12 knockdown enhanced c‐Myc binding to *IFN‐γ* and *GZMB* promoters while diminishing its binding to *KRAS* and *CDK4* promoters (Figure 3A,D). These results were validated by ChIP‐qPCR, which showed decreased c‐Myc enrichment on proliferation‐related gene promoters in MM cells following HDAC2 or circANKRD12 knockdown (Figure 3B,C). Conversely, ChIP‐qPCR detected increased c‐Myc enrichment on effector gene promoters in NK cells after HDAC2 or circANKRD12 knockdown (Figure 3E,F). Although c‐Myc does not directly act as an oncogene in NK cells, its suppressive effect on NK cell function promotes tumor immune evasion. Thus, ac4C modification facilitates myeloma progression and NK cell dysfunction through distinct c‐Myc‐mediated transcriptional mechanisms in MM and NK cells.

### 3.4 CircANKRD12 Is Upregulated in MM Patients and Drives MM Proliferation In Vitro and In Vivo

**FIGURE 4:**
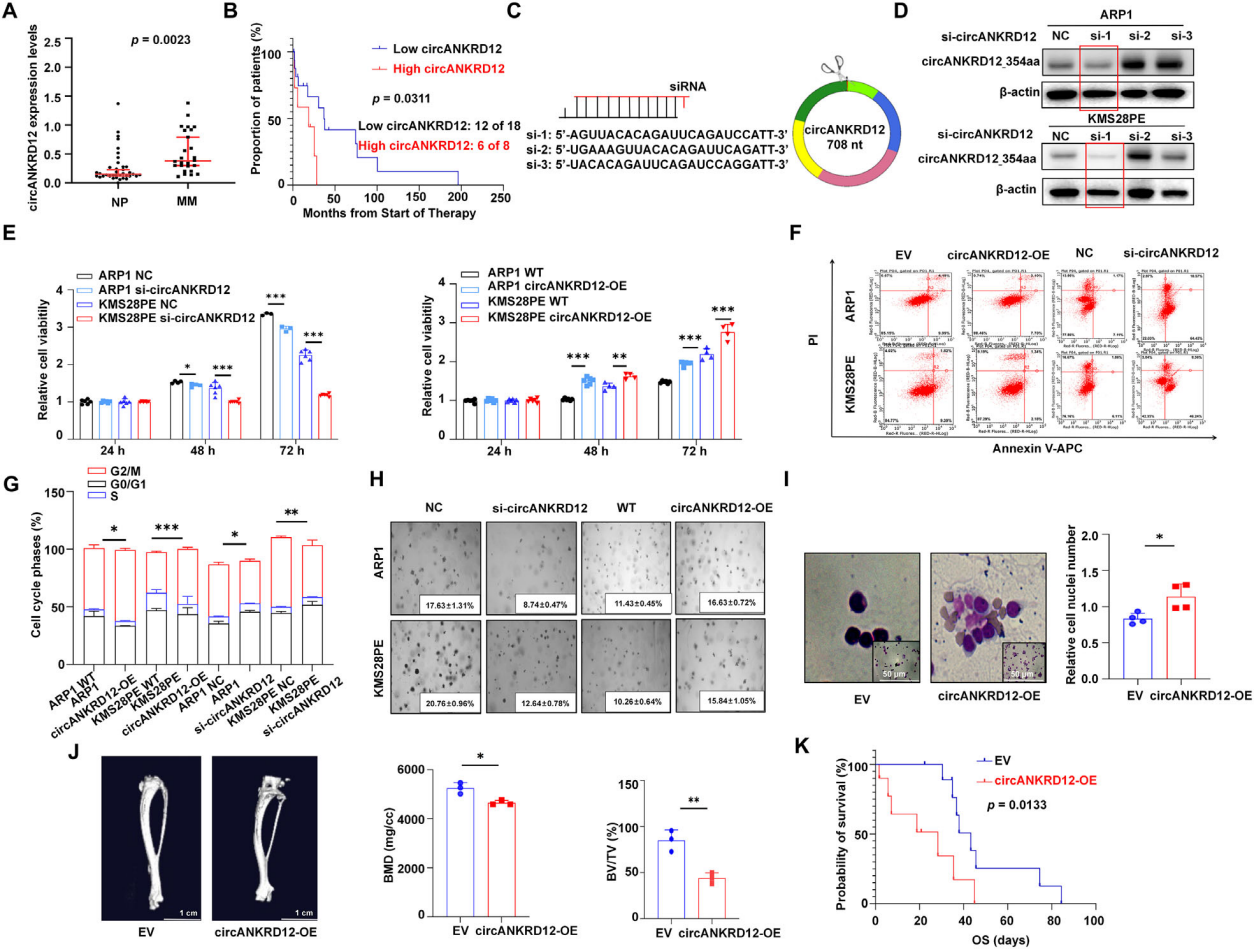
CircANKRD12 is upregulated in MM patients and drives MM proliferation in vitro and in vivo. (A) CircANKRD12 level was significantly elevated in MM patients (*n* = 26). (B) High circANKRD12 expression was associated with shorter EFS. (C) Schematic of siRNA design targeting specific sequences of linear ANKRD12 and circANKRD12_354aa. (D) WB confirmation of circANKRD12_354aa knockdown in ARP1 and KMS28PE cells transfected with siRNA‐1 (si‐1). (E) CCK‐8 assay was used to detect the proliferation of ARP1 and KMS28PE cells upon si‐circANKRD12 treatment or circANKRD12 overexpression (*n* = 6). (F) Flow cytometry was used to detect apoptosis in ARP1 and KMS28PE cells upon si‐circANKRD12 treatment or circANKRD12 overexpression (*n* = 3). (G) Flow cytometry was used to detect the cell cycle of ARP1 and KMS28PE cells upon si‐circANKRD12 treatment or circANKRD12 overexpression (*n* = 3). (H) Soft agar cloning assay was used to examine the long‐term proliferation of ARP1 and KMS28PE cells upon si‐circANKRD12 treatment or circANKRD12 overexpression (*n* = 3). (I) Giemsa staining and quantitative analysis of bone marrow from pristane‐induced myeloma mice (*n* = 3). Scale bar: 50 µm. (J) Representative micro‐CT images and quantitative analysis of bone lesions in pristane‐induced myeloma mice (*n* = 3). Scale bar: 1 cm. (K) CircANKRD12 overexpression reduces survival in the pristane‐induced myeloma mouse model (*n* = 10). For comparisons between two groups, two‐tailed unpaired Student's *t*‐tests were used. Survival curves were analyzed using the log‐rank (Mantel–Cox) test. All data are displayed as mean ± SD; ^*^ *p* < 0.05, ^**^ *p* < 0.01, ^***^ *p* < 0.001.

To validate our findings in clinical samples, we analyzed blood samples from 26 patients with MM and 37 healthy individuals. We found that levels of circANKRD12 were significantly higher in MM patients (*p* < 0.01) (Figure 4A), and that increased expression was associated with a lower event‐free survival (EFS) rate (*p* < 0.0) (Figure 4B), indicating its potential as a biomarker for the progression of MM.

In MM cells, circANKRD12 was downregulated using specific siRNA (Figure 4C), which was further confirmed by WB analysis (Figure 4D). CCK‐8 assays demonstrated that overexpression of circANKRD12 significantly increased the proliferation rates of ARP1 and KMS28PE cells, while knockdown of circANKRD12 decreased their proliferation rates (Figure 4E). Flow cytometry analysis revealed that overexpression of circANKRD12 reduced, while knockdown increased, the rate of apoptosis in MM cells (*p <* 0.05) (Figure 4F). It also showed that overexpression of circANKRD12 increased, while knockdown decreased, the proportion of cells in the G2/M phase (*p* < 0.0) (Figure 4G). Clonogenic soft agar assays indicated that overexpression of circANKRD12 enhanced, while knockdown impaired, the long‐term self‐renewal capacity of MM cells, as reflected by colony formation (Figure 4H).

To further confirm our findings, we used a pristane‐induced model for spontaneous plasma cell tumors. At 16 weeks, we observed ascites development and abdominal distention, and Giemsa staining confirmed the successful establishment of the MM model (Figure 4I). Micro‐CT analysis showed a significant decrease in bone mineral density (BMD) and bone volume/trabeculae volume (BV/TV) in the circANKRD12‐OE group (Figure 4J). Ultimately, we found that overexpression of circANKRD12 significantly shortened mouse survival (Figure 4K) (Table S6) (*n* = 10). These in vitro and in vivo results confirm the role of circANKRD12 in promoting MM cell proliferation.

### 3.5 The Oncogenic Activity of circANKRD12 Requires ac4C Modification and Mediates HDAC2‐Dependent Proliferation

**FIGURE 5:**
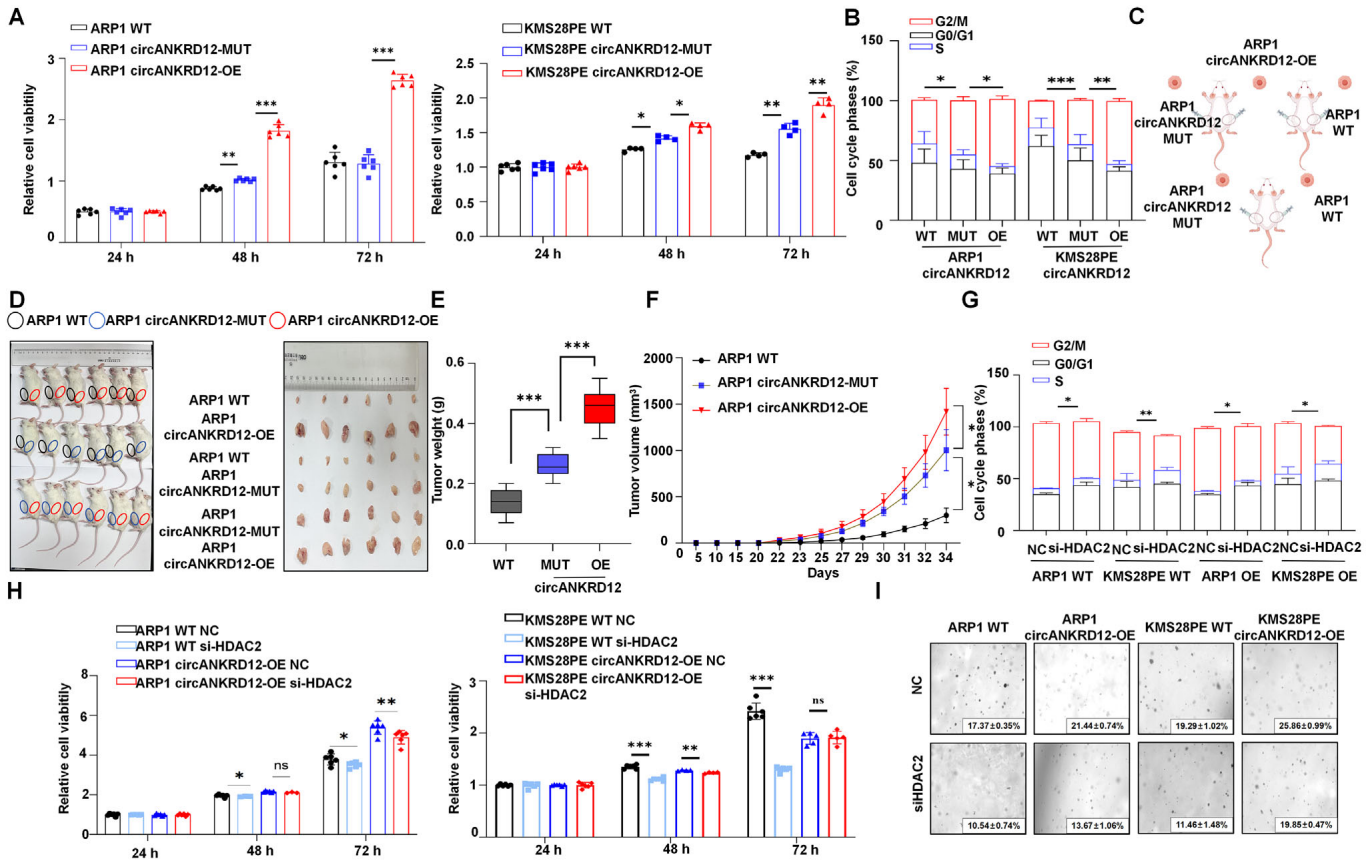
The oncogenic activity of circANKRD12 requires ac4C modification and mediates HDAC2‐dependent proliferation. (A) CCK‐8 assay indicated significantly reduced proliferation in circANKRD12‐MUT cells compared to circANKRD12‐OE cells (*n* = 6). (B) Cell cycle distribution was analyzed by flow cytometry in ARP1 and KMS28PE cells expressing circANKRD12‐MUT (*n* = 3). (C) Schematic diagram of the xenograft tumor model. (D) Representative photographs of tumor‐bearing mice at day 34; xenograft tumors from the indicated groups of NOD‐SCID mice were excised for further analysis. (E) Tumor weights were measured at day 34 post‐injection in the WT, circANKRD12‐OE, and circANKRD12‐MUT groups. (F) Tumor growth curves in NOD‐SCID mice (*n* = 6). (G) Flow cytometric analysis of the cell cycle in MM cells following si‐HDAC2 treatment (*n* = 3). (H) Knockdown of HDAC2 markedly inhibited MM cell proliferation as assessed by CCK‐8 assay (*n* = 6). (I) Long‐term proliferative capacity was evaluated by soft agar colony formation assay in ARP1 and KMS28PE cells after si‐HDAC2 treatment (*n* = 3). For comparisons between two groups, two‐tailed unpaired Student's *t*‐tests were used. All data are displayed as mean ± SD; ^*^ *p* < 0.05, ^**^ *p* < 0.01, ^***^ *p* < 0.001.

To determine whether circANKRD12 functions through ac4C modification, we examined the effects of mutating its ac4C site. CCK‐8 assays revealed that the proliferative capacity of circANKRD12‐MUT MM cells was reduced compared to circANKRD12‐OE cells (Figure 5A). Additionally, cell cycle analysis showed that the mutation attenuated the cell cycle‐promoting effect (Figure 5B). To validate these findings in vivo, WT, circANKRD12‐OE, and circANKRD12‐MUT cells were subcutaneously injected into NOD‐SCID mice (Figure 5C). Tumors derived from circANKRD12‐OE cells exhibited accelerated growth, with significantly greater weight and volume than those in the WT and MUT groups (*p <* 0.05) (Figure 5D–F). These results demonstrate that NAT10‐mediated acetylation of circANKRD12 enhances its translation efficiency and promotes MM proliferation.

Based on prior evidence, we hypothesized that circANKRD12 acts via HDAC2. Flow cytometry confirmed that si‐HDAC2 delayed cell cycle progression (Figure 5G). Additionally, CCK‐8 assays demonstrated that si‐HDAC2 suppressed MM cell proliferation (Figure 5H). Furthermore, clonogenic assays revealed that si‐HDAC2 significantly impaired long‐term self‐renewal capacity, as evidenced by decreased colony formation (Figure 5I). These findings suggest that circANKRD12 promotes MM progression through its ac4C modification and interaction with HDAC2.

### 3.6 CircANKRD12 Attenuates NK Cell Cytotoxicity via Its ac4C Modification and Interaction With HDAC2

**FIGURE 6:**
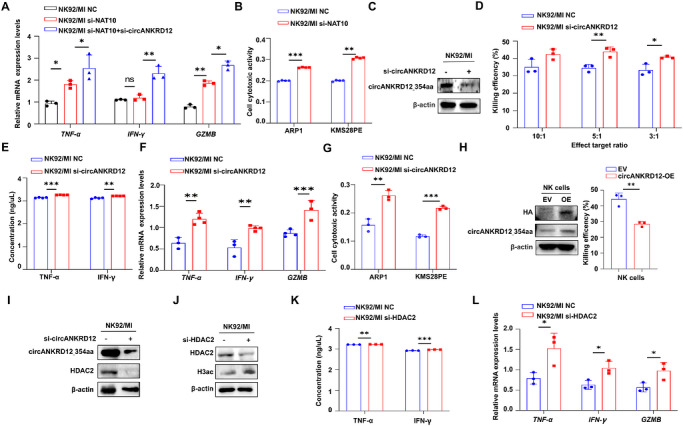
CircANKRD12 attenuates NK cell cytotoxicity via its ac4C modification and interaction with HDAC2. (A) The mRNA expression of effector factors in NK92/MI cells was measured using RT‐qPCR after si‐NAT10 and si‐circANKRD12 treatment (*n* = 3). (B) Cytotoxicity of NK92/MI cells after si‐NAT10 treatment, as assessed by LDH release assay (*n* = 6). (C) WB was used to detect the knockdown efficiency of si‐circANKRD12 in NK92/MI cells. (D) The killing efficiency of NK92/MI cells after si‐circANKRD12 treatment was detected (*n* = 6). (E) TNF‐α and IFN‐γ protein levels in NK92/MI cells after si‐circANKRD12 treatment, measured by ELISA (*n* = 6). (F) The mRNA expression levels of *TNF‐α*, *IFN‐γ*, and *GZMB* in NK92/MI cells after si‐circANKRD12 treatment were detected by RT‐qPCR (*n* = 6). (G) The cytotoxicity of NK92/MI cells after si‐circANKRD12 treatment was evaluated by LDH release assay (*n* = 3). (H) NK cells were isolated from the spleens of AAV‐infected, pristane‐induced myeloma mice. HA expression was verified by WB, and NK cell killing efficiency was determined. (I) WB analysis of HDAC2 expression in NK92/MI cells after si‐circANKRD12 treatment. (J) WB detection of HDAC2 and H3ac expression in NK92/MI cells after si‐HDAC2 treatment. (K) TNF‐α and IFN‐γ levels in NK92/MI cells after si‐HDAC2 treatment were measured by ELISA (*n* = 3). (L) The mRNA expression levels of *TNF‐α*, *IFN‐γ*, and *GZMB* in NK92/MI cells after si‐HDAC2 treatment were analyzed by RT‐qPCR (*n* = 6). For comparisons between two groups, two‐tailed unpaired Student's *t*‐tests were used. All data are displayed as mean ± SD; ^*^ *p* < 0.05, ^**^ *p* < 0.01, ^***^ *p* < 0.001, n.s. for no significance.

RT‐qPCR analysis showed that si‐NAT10 increased the mRNA levels of *TNF‐α*, *IFN‐γ*, and *GZMB* in NK92/MI cells, an effect that was further enhanced by concurrent knockdown of circANKRD12 (Figure 6A). LDH release assays confirmed that si‐NAT10 also increased the cytotoxicity of NK cells against tumor cells (Figure 6B), indicating that the immunosuppressive role of NAT10 in NK cells is mediated through circANKRD12. Furthermore, knockdown of circANKRD12 in NK92/MI cells significantly enhanced their cytotoxicity and cytokine release against MM cells, particularly at an effector‐to‐target ratio of 5:1 (Figure 6C,D). RT‐qPCR and ELISA analyses revealed that circANKRD12 knockdown significantly elevated both the expression and secretion of TNF‐α, IFN‐γ, and GZMB (Figure 6E,F). Consistently, LDH assays showed increased NK cell cytotoxicity following circANKRD12 knockdown (Figure 6G).

We used CD49b magnetic beads to isolate NK cells from AAV‐infected MM mice, yielding NK circANKRD12‐OE cells. The in vitro killing assays revealed that the tumor‐killing ability of NK circANKRD12‐OE cells was significantly impaired compared to that of WT NK cells (Figure 6H). We then investigated the circANKRD12‐HDAC2/c‐Myc pathway in NK92/MI cells. Silencing of circANKRD12 led to a decrease in HDAC2 expression (Figure 6I). Similarly, si‐HDAC2 reduced HDAC2 levels and increased H3ac levels (Figure 6J). RT‐qPCR and ELISA analyses confirmed that si‐HDAC2 significantly increased the transcription of NK cell effector factors (Figure 6K,L), identifying HDAC2 as a functional inhibitor of NK cell cytotoxicity. These findings demonstrate that the anti‐cytotoxic effect of circANKRD12 on NK cells is mediated by its ac4C modification and its interaction with HDAC2.

### 3.7 CircANKRD12 Fuels MM Malignant Progression Through Intercellular Transfer Between MM and NK Cells

**FIGURE 7:**
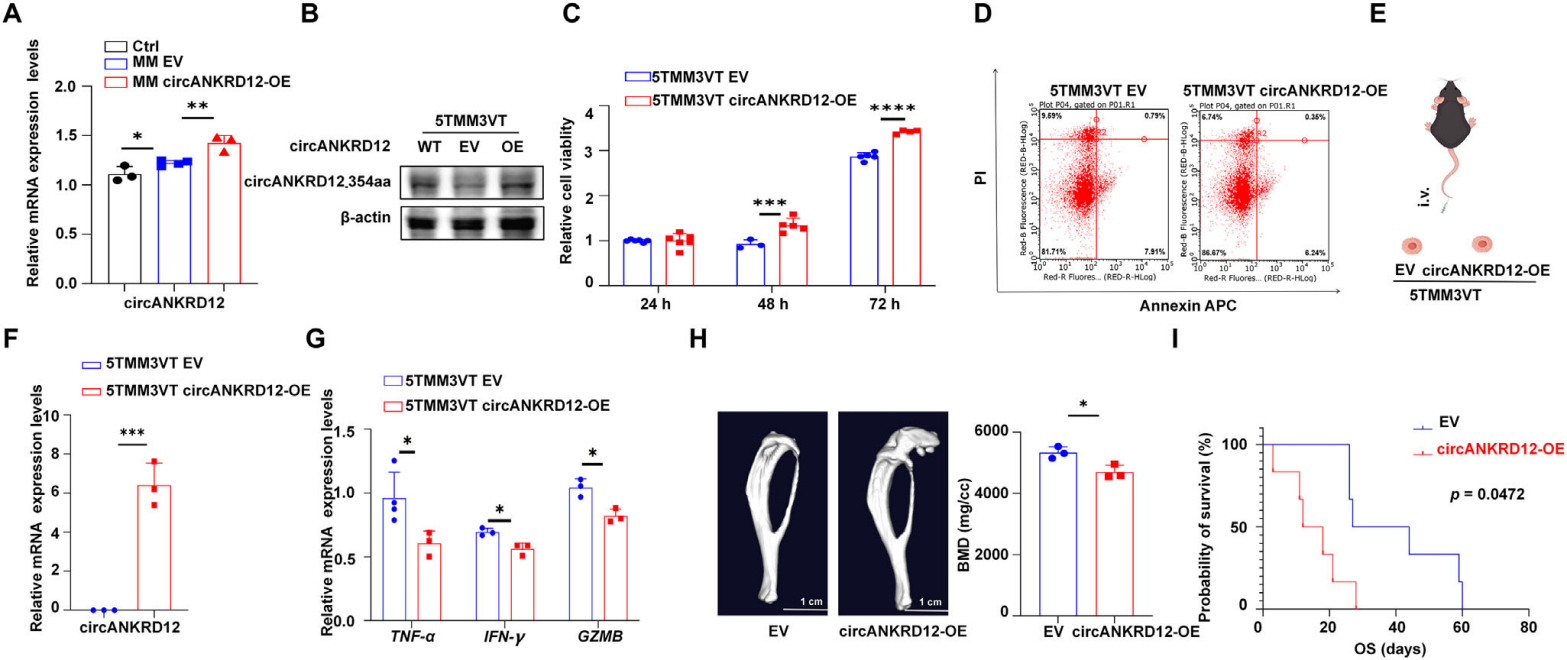
CircANKRD12 fuels MM malignant progression through intercellular transfer between MM and NK cells. (A) RT‐qPCR analysis of circANKRD12 levels in serum from AAV‐infected, pristane‐induced myeloma mice (*n* = 4). (B) Construction of 5TMM3VT circANKRD12‐OE cells using the human circANKRD12 sequence. (C) CCK‐8 was used to detect the proliferation of 5TMM3VT circANKRD12‐OE cells (*n* = 6). (D) Flow cytometry was used to detect apoptosis of 5TMM3VT circANKRD12‐OE cells (*n* = 3). (E) Schematic diagram of the 5TMM3VT mouse model. (F) RT‐qPCR was used to detect human circANKRD12 expression in NK cells from 5TMM3VT model mice (*n* = 3). (G) RT‐qPCR was used to detect the expression of effector factors in NK cells from 5TMM3VT model mice (*n* = 5). (H) Representative micro‐CT images and quantitative analysis of bone lesions in 5TMM3VT model mice (*n* = 3). Scale bar: 1 cm. (I) Survival curve of 5TMM3VT model mice (*n* = 6). For comparisons between two groups, two‐tailed unpaired Student's *t*‐tests were used. Survival curves were analyzed using the log‐rank (Mantel–Cox) test. All data are displayed as mean ± SD; ^*^ *p* < 0.05, ^**^ *p* < 0.01, ^***^ *p* < 0.001, ^****^ *p* < 0.0001.

RT‐qPCR analysis of serum from MM model mice revealed that levels of circANKRD12 were significantly higher in the circANKRD12‐OE group compared to the WT group (Figure 7A), indicating that circANKRD12 is secreted by MM cells into the microenvironment. To further investigate the impact of tumor‐derived circANKRD12 on NK cells, we established a 5TMM3VT mouse model using cells stably expressing human circANKRD12 (Figure 7B). Phenotypic analysis confirmed that human circANKRD12 promoted the proliferation of 5TMM3VT cells and inhibited their apoptosis (Figure 7C,D). These cells were then injected into C57BL/KaLwRij mice to construct the 5TMM3VT MM mouse model (Figure 7E). After 2 weeks, NK cells were isolated and found to contain detectable levels of human circANKRD12 (Figure 7F), which resulted in impaired function (Figure 7G). In the 5TMM3VT model, elevated levels of circANKRD12 exacerbated bone lesions and reduced survival compared to controls (Figure 7H,I, Table S7, *n* = 6). These results demonstrate that circANKRD12 is secreted by MM cells, enters immune cells, and suppresses their function.

### 3.8 Desloratadine Suppresses MM and Augments NK Cell Activity via circANKRD12_354aa Inhibition

**FIGURE 8:**
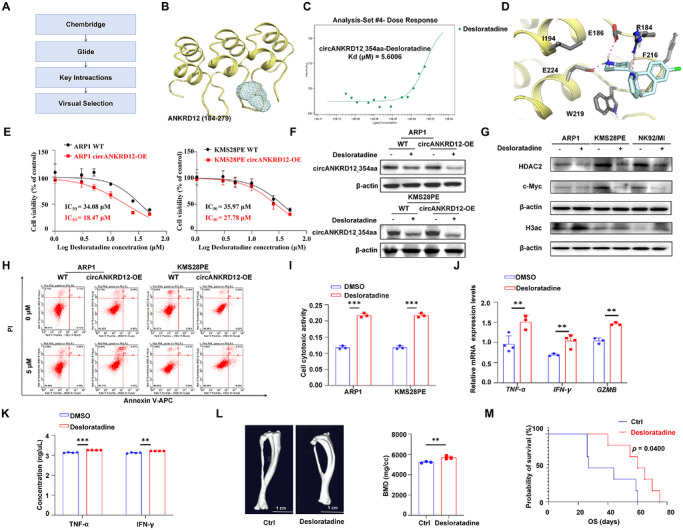
Desloratadine suppresses MM and augments NK cell activity via circANKRD12_354aa inhibition. (A) Schematic of the structure‐based virtual screening workflow. (B) Predicted structure (AlphaFold) of the ANKRD12 fragment (residues 184–279), shown in pale yellow cartoon representation, with its putative binding pocket outlined by a pale blue mesh. (C) Interaction between circANKRD12_354aa protein and desloratadine, analyzed by MST. (D) Detailed view of desloratadine (pale blue sticks) bound within the ANKRD12 pocket. Interacting residues are shown as gray sticks, with key contacts indicated by magenta dashed lines. (E) IC_50_ of desloratadine in MM cells determined by MTT assay (*n* = 6). (F) WB analysis of circANKRD12_354aa protein inhibition by desloratadine in MM cells. (G) Effect of desloratadine on the HDAC2/c‐Myc axis in MM cells assessed by WB. (H) Effect of desloratadine on MM cell apoptosis analyzed by flow cytometry (*n* = 3). (I) Cytotoxic effect of NK92/MI cells on MM cells following desloratadine treatment (*n* = 6). (J) The mRNA expression levels of *TNF‐α*, *IFN‐γ*, and *GZMB* in NK92/MI cells treated with desloratadine, measured by RT‐qPCR (*n* = 6). (K) TNF‐α and IFN‐γ levels in NK92/MI cells after desloratadine treatment were measured by ELISA (*n* = 3). (L) Representative micro‐CT images and quantitative analysis of bone lesions in 5TMM3VT model mice treated with desloratadine (*n* = 3). Scale bar: 1 cm. (M) Survival curve of 5TMM3VT model mice (*n* = 6). For comparisons between two groups, two‐tailed unpaired Student's *t*‐tests were used. Survival curves were analyzed using the log‐rank (Mantel–Cox) test. All data are displayed as mean ± SD; ^*^ *p* < 0.05, ^**^ *p* < 0.01, ^***^ *p* < 0.001.

As no crystal structure or small‐molecule ligand for ANKRD12 has been reported, we conducted high‐throughput computational screening to identify compounds that could target circANKRD12_354aa (Figure 8A). Using an AlphaFold‐predicted model (residues 184–279), we identified a potential ligand‐binding pocket (Figure 8B). Through virtual screening, we identified desloratadine as a potential binder, and further confirmed its binding affinity (KD) of 5.6006 µm through MST assays (Figure 8C). Docking analysis revealed that the protonated piperidine nitrogen of desloratadine forms hydrogen‐bond/ionic interactions with E86 and E224, while the pyridine‐ring nitrogen accepts a hydrogen‐bond from R184 (Figure 8D).

We conducted further research to determine the potential therapeutic effects of targeting circANKRD12_354aa with desloratadine. CCK‐8 assays showed that circANKRD12‐OE cells were more sensitive to desloratadine, exhibiting a significantly lower IC_50_ than WT cells (Figure 8E). WB analysis also confirmed that desloratadine decreased levels of circANKRD12_354aa protein (Figure 8F). Additionally, treatment with desloratadine resulted in downregulation of HDAC2 and c‐Myc expression, as well as an increase in H3ac levels (Figure 8G). Flow cytometry analysis further demonstrated an increase in apoptosis in MM cells following desloratadine treatment (Figure 8H).

To evaluate the effect of desloratadine on NK cells, we performed RT‐qPCR, LDH release, and ELISA assays, which collectively demonstrated improved NK cell function after treatment (Figure 8I–K). In the 5TMM3VT model, desloratadine administration significantly improved bone density and extended survival compared to untreated controls (Figure 8L,M). These findings suggest that targeting circANKRD12 with desloratadine can effectively suppress the progression of MM and enhance NK cell‐mediated immune function, highlighting circANKRD12 as a promising therapeutic target.

## 4 Discussion

The persistent challenge of relapse in MM highlights the critical need to identify new molecular drivers that promote both tumor proliferation and immune evasion [24, 25]. Our study addresses this urgent need by uncovering a previously unknown oncogenic pathway involving NAT10‐mediated acetylation of circANKRD12. We demonstrate that NAT10 catalyzes the acetylation of circANKRD12, leading to its stabilization and translation into a novel 354‐amino acid protein (circANKRD12_354aa). CircANKRD12_354aa orchestrates tumor progression through two complementary mechanisms: in MM cells, it binds to HDAC2, stabilizing c‐Myc and promoting proliferation; simultaneously, it is transferred to NK cells, where it suppresses their cytotoxic function. Importantly, we identified the clinically used antihistamine desloratadine as a direct inhibitor of this protein, which in preclinical models potently suppressed MM growth and reinvigorated NK cell‐mediated killing.

Our research significantly expands the functional repertoire of NAT10. Beyond its established roles in ribosomal biogenesis [26, 27, 28] and mRNA translation [29], we reveal its crucial function in regulating circRNA modification and translation. Although NAT10 is implicated in MM progression through mechanisms such as *CEP170* mRNA acetylation, its broad expression in immune cells, including T, B, and NK cells, suggests a broader immunoregulatory capacity, as previously suggested by its role in inhibiting T cell function within the TME [15]. Our work extends this paradigm by demonstrating that NAT10's catalytic activity directly targets circRNAs. The identification of site‐specific ac4C modification on circANKRD12 as a critical driver of its translation unveils a new layer of post‐transcriptional control governing non‐canonical protein expression [30], suggesting that the NAT10‐circRNA axis may represent a widespread mechanism across cancer types. On the other hand, it is noteworthy that other ac4C sites may participate in the regulation of circANKRD12 under specific conditions (Figure S2), a finding warranting further investigation in the future.

The core effector of this axis is circANKRD12_354aa, which functions as a molecular scaffold that recruits HDAC2 to amplify c‐Myc oncogenic signaling. Our data demonstrate that this partnership leads to the deacetylation of c‐Myc, resulting in its hyperactivation, inhibition of K48‐linked ubiquitination, and subsequent stabilization [21]. HDAC2, a class I deacetylase, is a well‐established regulator of tumorigenesis, cell cycle progression, and immune evasion [31]. Its overexpression is associated with metastasis, aggressiveness, and poor prognosis in various cancers [32]. Furthermore, HDAC2 plays a role in modulating interferon signaling [33, 34, 35] and regulating immune checkpoints, such as PD‐L1, in tumor cells [36]. Our work identifies circANKRD12_354aa as a novel cofactor for HDAC2, specifically enhancing c‐Myc's transcriptional efficiency in MM cells.

c‐Myc alterations are common in myeloma and other cancers, driven by diverse genetic, epigenetic, and post‐translational mechanisms [37, 38, 39, 40]. As a master regulator, c‐Myc promotes cell cycle progression and transformation [41], and its dysregulation is frequently observed in MM [42]. c‐Myc activation induces the overexpression of key oncogenic genes, including *TP53*, *CDKN2A*, and *BCL2* [43, 44, 45]. Moreover, c‐Myc expression in cancer cells actively shapes the TME to support progression and immune dysfunction by affecting various immune cells (e.g., T cells, macrophages, NK cells), cytokines (e.g., TGFβ, IFNs), and immunomodulatory molecules (e.g., PD‐L1, CD47, MHC I) [46, 47, 48, 49, 50]. Notably, c‐Myc activity within tumor‐infiltrating immune cells can impair anti‐tumor immunity; for instance, it suppresses NK cell surveillance by repressing the STAT1/STAT2 and type I interferon pathways [51, 52, 53]. Our work unifies these observations by positioning the NAT10/circANKRD12/HDAC2 axis as an upstream regulator that coordinately drives c‐Myc's dual role in promoting both tumor cell proliferation and immune suppression. The distinct phenotypes arise from a fundamental divergence in c‐Myc's function: it drives a proliferative program in MM cells, yet suppresses effector functions in NK cells. The discovery that circANKRD12 can be shuttled via exosomes within the TME further underscores its potential as a druggable target to simultaneously disrupt MM proliferation and restore NK cell function. Although NAT10 is implicated in MM Although NAT10 is implicated in MM.

The translational potential of our study is highlighted by the identification of desloratadine as an inhibitor of circANKRD12_354aa. This clinically used antihistamine effectively binds and inhibits the oncogenic protein, disrupting the NAT10/circANKRD12/HDAC2/c‐Myc axis. In our models, desloratadine effectively suppressed MM proliferation and reactivated NK cell‐mediated killing in vivo. This dual effect positions the targeting of circANKRD12 as a promising strategy to simultaneously attack the tumor and remodel the immunosuppressive TME. However, translating this potential into clinical benefit requires further steps: rigorous clinical validation is essential to confirm efficacy in MM patients, and future medicinal chemistry efforts should focus on optimizing desloratadine‐derived compounds to enhance potency and minimize off‐target effects.

Several limitations of this study should be acknowledged. First, our clinical analysis of circANKRD12 was based on a relatively small cohort derived from a single center, which may not fully represent the broader patient population with multiple myeloma and could introduce selection bias. To address these limitations, we plan to validate our findings in future studies using larger, multi‑center cohorts that encompass a wider spectrum of myeloma subtypes and treatment backgrounds. Such validation will be essential to confirm the clinical relevance and generalizability of circANKRD12 as a potential biomarker. Second, while desloratadine exerts its immunomodulatory effects on NK cells primarily by targeting the circANKRD12_354aa protein, a systematic assessment of its target specificity is currently lacking. In future studies, orthogonal validation approaches such as CETSA, target gene knockout, or thermal proteome profiling will be employed to further corroborate the direct target engagement and functional specificity of desloratadine toward circANKRD12_354aa.

In summary, our study reveals that NAT10‐mediated ac4C modification of circANKRD12 promotes MM progression via the HDAC2/c‐Myc axis. Specifically, the translated product circANKRD12_354aa stabilizes c‐Myc, driving tumor cell proliferation while simultaneously suppressing NK cell cytotoxicity. Moreover, circANKRD12 can be transferred between MM cells and immune cells within the bone marrow microenvironment. Together, these findings establish circANKRD12 as a promising dual‐function therapeutic target, providing a strategy to concurrently suppress tumor growth and restore anti‐tumor immunity.

## Author Contributions

**Y.Y**. and **C.Y.G**. conceived and designed the study. **J.L.Z**. and **H.S**. carried out the experiments and analyzed the data. **C.W**. performed the 10X Genomics sequencing analysis. **C.Y.G**. and **J.L.Z**. drafted the manuscript. **C.W**., **Z.H.L**., and **L.X.Z**. contributed to methodology and visualization. **X.Y.L**. and **M.J.G**. were involved in formal analysis and validation. All authors have reviewed, approved the final version of the manuscript, and agreed to its submission.

## Funding

This work was supported by Climbing Plan of Basic Research Priorities Program of Jiangsu Province (BK20240003); the National Natural Science Foundation of China (82373936); a project funded by the Priority Academic Program Development of Jiangsu Higher Education Institutions (Traditional Chinese Medicine; Integration of Chinese and Western Medicine).

## Ethics Statement

The study received approval from the Ethics Committee of the Institutional Animal Care and Use Committee (IACUC) of Nanjing University of Chinese Medicine. Informed consent was obtained from all participants prior to their inclusion in the study. The approved animal ethics number is 202406A031.

## Consent

All authors have provided their consent for the publication of this manuscript.

## Conflicts of Interest

The authors declare no conflicts of interest.

## Supporting information




**Supporting File**: advs75797‐sup‐0001‐SuppMat.docx.

## Data Availability

The data that support the findings of this study are available from the corresponding author upon reasonable request.

## References

[1] M. Hu , W. Zhou , Y. Wang , et al., “Discovery of the First Potent Proteolysis Targeting Chimera (PROTAC) Degrader of Indoleamine 2,3‐Dioxygenase 1,” Acta Pharmaceutica Sinica B 10 (2020): 1943–1953, 10.1016/j.apsb.2020.02.010. 33163345 PMC7606109

[2] B. Ruf , B. Heinrich , and T. F. Greten , “Immunobiology and Immunotherapy of HCC: Spotlight on Innate and Innate‐Like Immune Cells,” Cellular & Molecular Immunology 18 (2020): 112–127, 10.1038/s41423-020-00572-w. 33235387 PMC7852696

[3] H. Xie , K. Zhang , H. Yin , et al., “Acetyltransferase NAT10 inhibits T‐cell Immunity and Promotes Nasopharyngeal Carcinoma Progression Through DDX5/HMGB1 Axis,” Journal for ImmunoTherapy of Cancer 13, no. 2 (2025): 010301, 10.1136/jitc-2024-010301. PMC11822433 39939141

[4] R. Liu , Y. Li , A. Wu , et al., “Identification of Plasma hsa_circ_0005397 and Combined With Serum AFP, AFP‐L3 as Potential Biomarkers for Hepatocellular Carcinoma,” Frontiers in Pharmacology 12 (2021): 639963, 10.3389/fphar.2021.639963. 33679420 PMC7933497

[5] C. Gu , W. Wang , X. Tang , et al., “CHEK1 and circCHEK1_246aa Evoke Chromosomal Instability and Induce Bone Lesion Formation in Multiple Myeloma,” Molecular Cancer 20 (2021): 84, 10.1186/s12943-021-01380-0. 34090465 PMC8178856

[6] T. Lan , F. Gao , Y. Cai , et al., “The Protein circPETH‐147aa Regulates Metabolic Reprogramming in Hepatocellular Carcinoma Cells to Remodel Immunosuppressive Microenvironment,” Nature Communications 16 (2025): 333, 10.1038/s41467-024-55577-0. PMC11696079 39747873

[7] X. Tang , Z. Deng , P. Ding , et al., “A Novel Protein Encoded by circHNRNPU Promotes Multiple Myeloma Progression by Regulating the Bone Marrow Microenvironment and Alternative Splicing,” Journal of Experimental & Clinical Cancer Research 41 (2022): 85, 10.1186/s13046-022-02276-7. 35260179 PMC8903708

[8] X. Tang , M. Guo , P. Ding , et al., “BUB1B and circBUB1B_544aa Aggravate Multiple Myeloma Malignancy Through Evoking Chromosomal Instability,” Signal Transduction and Targeted Therapy 6 (2021): 361, 10.1038/s41392-021-00746-6. 34620840 PMC8497505

[9] J. Fu , F. Liu , S. Bai , et al., “Circular RNA CDYL Facilitates Hepatocellular Carcinoma Stemness and PD‐L1^+^ Exosomes‐Mediated Immunotherapy Resistance via Stabilizing Hornerin Protein by Blocking Synoviolin 1‐Mediated Ubiquitination,” International Journal of Biological Macromolecules 310 (2025): 143246, 10.1016/j.ijbiomac.2025.143246. 40250664

[10] Z. Miao , J. Li , Y. Wang , et al., “Hsa_circ_0136666 Stimulates Gastric Cancer Progression and Tumor Immune Escape by Regulating the miR‐375/PRKDC Axis and PD‐L1 Phosphorylation,” Molecular Cancer 22 (2023): 205, 10.1186/s12943-023-01883-y. 38093288 PMC10718020

[11] X. Wan , L. Wang , M. A. Khan , et al., “NAT10‐Mediated N4‐Acetylcytidine Modification in KLF9 mRNA Promotes Adipogenesis,” Cell Death & Differentiation 32 (2025): 1613–1629, 10.1038/s41418-025-01483-x. 40123006 PMC12432206

[12] Z. Sun , Y. Wang , C. Zheng , et al., “NAT10 Promotes the Progression of Clear Cell Renal Cell Carcinoma by Regulating ac4C Acetylation of NFE2L3 and Activating AKT/GSK3β Signaling Pathway,” Cell Death & Disease 16 (2025): 235, 10.1038/s41419-025-07528-w. 40169553 PMC11962090

[13] Q. Yang , X. Lei , J. He , et al., “N4‐Acetylcytidine Drives Glycolysis Addiction in Gastric Cancer via NAT10/SEPT9/HIF‐1 α Positive Feedback Loop,” Advanced Science 10 (2023): 2300898, 10.1002/advs.202300898. 37328448 PMC10427357

[14] R. Wei , X. Cui , J. Min , et al., “NAT10 Promotes Cell Proliferation by Acetylating CEP170 mRNA to Enhance Translation Efficiency in Multiple Myeloma,” Acta Pharmaceutica Sinica B 12 (2022): 3313–3325, 10.1016/j.apsb.2022.01.015. 35967285 PMC9366180

[15] S. Ito , S. Horikawa , T. Suzuki , et al., “Human NAT10 Is an ATP‐Dependent RNA Acetyltransferase Responsible for N4‐Acetylcytidine Formation in 18 S Ribosomal RNA (rRNA),” Journal of Biological Chemistry 289 (2014): 35724–35730, 10.1074/jbc.C114.602698. 25411247 PMC4276842

[16] Z. Deng , S. Sun , N. Zhou , et al., “PNPO‐Mediated Oxidation of DVL3 Promotes Multiple Myeloma Malignancy and Osteoclastogenesis by Activating the Wnt/β‐Catenin Pathway,” Advanced Science 12, no. 5 (2024): 2407681, 10.1002/advs.202407681. 39656865 PMC11792023

[17] W. Zhao , Y. Zhou , Q. Cui , and Y. Zhou , “PACES: Prediction of N4‐Acetylcytidine (ac4C) Modification Sites in mRNA,” Scientific Reports 9 (2019): 11112, 10.1038/s41598-019-47594-7. 31366994 PMC6668381

[18] M. Zrimšek , K. Draganić , A. Malzer , et al., “HDAC1 Acts as a Tumor Suppressor in ALK‐Positive Anaplastic Large Cell Lymphoma: Implications for HDAC Inhibitor Therapy: Lymphoma,” Leukemia 39 (2025): 1412–1424. 40175628 10.1038/s41375-025-02584-9 PMC12133565

[19] A. Zhang , P. L. Yeung , C.‐W. Li , et al., “Identification of a Novel Family of Ankyrin Repeats Containing Cofactors for p160 Nuclear Receptor Coactivators,” Journal of Biological Chemistry 279 (2004): 33799–33805, 10.1074/jbc.M403997200. 15184363

[20] D. Jiao , R. Sun , X. Ren , et al., “Lipid Accumulation‐Mediated Histone Hypoacetylation Drives Persistent NK Cell Dysfunction in Anti‐Tumor Immunity,” Cell Reports 43 (2024): 113632, 10.1016/j.celrep.2023.113632. 38117651

[21] Z. Wang , Y. Li , J. Yang , et al., “CircCFL1 Promotes TNBC Stemness and Immunoescape via Deacetylation‐Mediated c‐Myc Deubiquitylation to Facilitate Mutant TP53 Transcription,” Advanced Science 11, no. 34 (2024): 2404628, 10.1002/advs.202404628. 38981022 PMC11425638

[22] F. J. Gössl , P. Polo , F. Helmprobst , et al., “ER‐Phagy Mediates the Anti‐Tumoral Synergism Between HDAC Inhibition and Chemotherapy,” Cell Communication and Signaling 23, no. 1 (2025): 202. 40287668 10.1186/s12964-025-02198-9 PMC12034116

[23] S. Palfi , L. C. Peres , H. Koelmeyer , et al., “Cardiovascular Adverse Events and Outcomes After Anti‐BCMA CAR‐T for Relapsed and Refractory Multiple Myeloma,” Blood Cancer Journal 15 (2025): 63, 10.1038/s41408-025-01261-5. 40229239 PMC11997198

[24] D. Zhou , Q. Sun , J. Xia , et al., “Anti‐BCMA/GPRC5D Bispecific CAR T Cells in Patients with Relapsed or Refractory Multiple Myeloma: A Single‐Arm, Single‐Centre, Phase 1 Trial,” The Lancet Haematology 11 (2024): e751–e760, 10.1016/S2352-3026(24)00176-5. 39059405

[25] H.‐Y. Liu , Y.‐Y. Liu , F. Yang , et al., “Acetylation of MORC2 by NAT10 Regulates Cell‐Cycle Checkpoint Control and Resistance to DNA‐Damaging Chemotherapy and Radiotherapy in Breast Cancer,” Nucleic Acids Research 48 (2020): 3638–3656, 10.1093/nar/gkaa130. 32112098 PMC7144926

[26] J. Liu , Z. Gu , L. Zou , et al., “Acetyltransferase NAT10 Promotes an Immunosuppressive Microenvironment by Modulating CD^8+^ T Cell Activity in Prostate Cancer,” Molecular Biomedicine 5 (2024): 67, 10.1186/s43556-024-00228-5. 39648231 PMC11625704

[27] X. Liu , Y. Tan , C. Zhang , et al., “NAT10 Regulates p53 Activation Through Acetylating p53 at K120 and Ubiquitinating Mdm2,” The EMBO Reports 17 (2016): 349–366, 10.15252/embr.201540505. 26882543 PMC4772976

[28] D. Dominissini and G. N. Rechavi , “N4‐acetylation of Cytidine in mRNA by NAT10 Regulates Stability and Translation,” Cell 175 (2018): 1725–1727, 10.1016/j.cell.2018.11.037. 30550783

[29] A. Castello , B. Fischer , K. Eichelbaum , et al., “Insights Into RNA Biology From an Atlas of Mammalian mRNA‐Binding Proteins,” Cell 149 (2012): 1393–1406, 10.1016/j.cell.2012.04.031. 22658674

[30] X. Zhu , X. Zhang , Y. Qin , et al., “Circular RNA circATM Binds PARP1 to Suppress Wnt/β‐Catenin Signaling and Induce Cell Cycle Arrest in Gastric Cancer Cells,” Journal of Advanced Research 80 (2025): 609–625. 40288674 10.1016/j.jare.2025.04.033 PMC12869237

[31] O. H. Krämer , “HDAC2: A Critical Factor in Health and Disease,” Trends in Pharmacological Sciences 30 (2009): 647–655, 10.1016/j.tips.2009.09.007. 19892411

[32] W. Shan , Y. Jiang , H. Yu , et al., “HDAC2 Overexpression Correlates With Aggressive Clinicopathological Features and DNA‐Damage Response Pathway of Breast Cancer,” American journal of cancer research 7 (2017): 1213–1226. 28560068 PMC5446485

[33] L. Klampfer , J. Huang , L.‐A. Swaby , and L. Augenlicht , “Requirement of Histone Deacetylase Activity for Signaling by STAT1,” Journal of Biological Chemistry 279 (2004): 30358–30368, 10.1074/jbc.M401359200. 15123634

[34] L. Icardi , S. Lievens , R. Mori , et al., “Opposed Regulation of Type I IFN‐Induced STAT3 and ISGF3 Transcriptional Activities by Histone Deacetylases (HDACS) 1 and 2,” The FASEB Journal 26 (2011): 240–249, 10.1096/fj.11-191122. 21957129

[35] P. Xu , S. Ye , K. Li , et al., “NOS1 Inhibits the Interferon Response of Cancer Cells by S‐nitrosylation of HDAC2,” Journal of Experimental & Clinical Cancer Research 38 (2019): 483, 10.1186/s13046-019-1448-9. 31805977 PMC6896289

[36] Y. Gao , N. T. Nihira , X. Bu , et al., “Acetylation‐Dependent Regulation of PD‐L1 Nuclear Translocation Dictates the Efficacy of Anti‐PD‐1 Immunotherapy,” Nature Cell Biology 22 (2020): 1064–1075, 10.1038/s41556-020-0562-4. 32839551 PMC7484128

[37] N. Meyer and L. Z. Penn , “Reflecting on 25 years With MYC,” Nature Reviews Cancer 8 (2008): 976–990, 10.1038/nrc2231. 19029958

[38] F. X. Schaub , V. Dhankani , A. C. Berger , et al., “Pan‐Cancer Alterations of the MYC Oncogene and its Proximal Network Across the Cancer Genome Atlas,” Cell Systems 6 (2018): 282–300, 10.1016/j.cels.2018.03.003. 29596783 PMC5892207

[39] C. V. Dang , “MYC on the Path to Cancer,” Cell 149 (2012): 22–35. 22464321 10.1016/j.cell.2012.03.003 PMC3345192

[40] M. Kalkat , J. De Melo , K. Hickman , et al., “MYC Deregulation in Primary Human Cancers,” Genes 8 (2017): 151, 10.3390/genes8060151. 28587062 PMC5485515

[41] G. Yang and P. Hurlin , “MNT and Emerging Concepts of MNT‐MYC Antagonism,” Genes 8 (2017): 83, 10.3390/genes8020083. 28230739 PMC5333072

[42] J. Edelmann , E. Tausch , D. A. Landau , et al., “Frequent Evolution of Copy Number Alterations in CLL Following First‐Line Treatment with FC (R) Is Enriched with TP53 Alterations: Results from the CLL8 Trial,” Leukemia 31 (2016): 734–738, 10.1038/leu.2016.317. 27909343 PMC5332302

[43] S. T. Bailey , A. M. Smith , J. Kardos , et al., “MYC Activation Cooperates With Vhl and Ink4a/Arf Loss to Induce Clear Cell Renal Cell Carcinoma,” Nature Communications 8 (2017): 15770, 10.1038/ncomms15770. PMC5472759 28593993

[44] T. Teitz , T. Wei , M. B. Valentine , et al., “Caspase 8 is Deleted or Silenced Preferentially in Childhood Neuroblastomas With Amplification of MYCN,” Nature Medicine 6 (2000): 529–535, 10.1038/75007. 10802708

[45] D. L. Vaux , S. Cory , and J. M. Adams , “Bcl‐2 Gene Promotes Haemopoietic Cell Survival and Cooperates With c‐myc to Immortalize pre‐B Cells,” Nature 335 (1988): 440–442, 10.1038/335440a0. 3262202

[46] M. Reimann , S. Lee , C. Loddenkemper , et al., “Tumor Stroma‐Derived TGF‐β Limits Myc‐Driven Lymphomagenesis via Suv39h1‐Dependent Senescence,” Cancer Cell 17 (2010): 262–272, 10.1016/j.ccr.2009.12.043. 20227040

[47] Y. Xu , M. Poggio , H. Y. Jin , et al., “Translation Control of the Immune Checkpoint in Cancer and its Therapeutic Targeting,” Nature Medicine 25 (2019): 301–311, 10.1038/s41591-018-0321-2. PMC6613562 30643286

[48] R. Dhanasekaran , V. Baylot , M. Kim , et al., “MYC and Twist1 Cooperate to Drive Metastasis by Eliciting Crosstalk Between Cancer and Innate Immunity,” eLife 9 (2020): 50731. 10.7554/eLife.50731 PMC6959993 31933479

[49] J. van Riggelen , J. Müller , T. Otto , et al., “The Interaction Between Myc and Miz1 is Required to Antagonize TGFβ‐Dependent Autocrine Signaling During Lymphoma Formation and Maintenance,” Genes & Development 24 (2010): 1281–1294, 10.1101/gad.585710. 20551174 PMC2885663

[50] R. L. Vartuli , H. Zhou , L. Zhang , et al., “Eya3 Promotes Breast Tumor–Associated Immune Suppression via Threonine Phosphatase–Mediated PD‐L1 Upregulation,” Journal of Clinical Investigation 128 (2018): 2535–2550, 10.1172/JCI96784. 29757193 PMC5983346

[51] N. Muthalagu , T. Monteverde , X. Raffo‐Iraolagoitia , et al., “Repression of the Type I Interferon Pathway Underlies MYC‐ and KRAS‐Dependent Evasion of NK and B Cells in Pancreatic Ductal Adenocarcinoma,” Cancer Discovery 10 (2020): 872–887, 10.1158/2159-8290.CD-19-0620. 32200350 PMC7611248

[52] S. Swaminathan , A. S. Hansen , L. D. Heftdal , et al., “MYC Functions as a Switch for Natural Killer Cell‐Mediated Immune Surveillance of Lymphoid Malignancies,” Nature Communications 11 (2020): 2860, 10.1038/s41467-020-16447-7. PMC7275060 32503978

[53] R. M. Kortlever , N. M. Sodir , C. H. Wilson , et al., “Myc Cooperates With Ras by Programming Inflammation and Immune Suppression,” Cell 171 (2017): 1301–1315, 10.1016/j.cell.2017.11.013. 29195074 PMC5720393

