# Supplementary material for: NAT10‐Mediated ac4C Modification of circANKRD12 Reprograms the Tumor Microenvironment
Source: Adv Sci (Weinh). 2026 May 22:e75797. Online ahead of print. doi: 10.1002/advs.75797 (PMC13336040; doi:10.1002/advs.75797)
Supplement: Supplementary file 1 — Supporting File: advs75797‐sup‐0001‐SuppMat.docx. [file ADVS-9999-e75797-s001.docx]

Supplementary Figure and Tables

NAT10-mediated ac4C Modification of circANKRD12 Reprograms the Tumor Microenvironment

Jiale Zhang^1,2^*, Hui Shi^2^*, Chen Wang^2,^, Zihao Liu^2^, Lianxin Zhou^2^, Xinyu Lv^2^, Mengjie Guo^2^, Chunyan Gu^1,2^, Ye Yang^2^

Supplementary Tables

Table S1 Sequences of small interfering RNA

| Name | | Sequence (5’-3’) |
| --- | --- | --- |
| si-HDAC2 | Sense | *GUCCUUCAAACAUGACAAATT* |
|  | Antisense | *UUUGUCAUGUUUGAAGGACTT* |
| si-NAT10 | Sense | *GCAUGGACCUCUCUGAAUATT* |
|  | Antisense | *UAUUCAGAGAGGUCCAUGCTT* |
| si-circANKRD12 | Sense | *UACACAGAUUCAGAUCCAGGATT* |
|  | Antisense | *UCCUGGAUCUGAAUCUGUGUATT* |
| Negative Control (NC) | Sense | *UUCUCCGAACGUGUCACGUTT* |
|  | Antisense | *ACGUGACACGUUCGGAGAATT* |

Table S2 Primer sequences for RT-qPCR

| Name | | Sequence (5’-3’) |
| --- | --- | --- |
| h-circANKRD12 | Forward primer | *CTAGCTAGCATTCAGATCCAGGACATACA* |
|  | Reverse primer | *CCGCTCGAGCTGTGTAACTTTCATCATCA* |
| h-IFN-γ | Forward primer | *GAGTGTGGAGACCATCAAGGAAG* |
|  | Reverse primer | *GGCGACAGTTCAGCCATCAC* |
| h-TNF-α | Forward primer | *AGCCCATGTTGTAGCAAACC* |
|  | Reverse primer | *TGAGGTACAGGCCCTCTGAT* |
| m-TNF-α | Forward primer | *CCCTCACACTCAGATCATCTTCT* |
|  | Reverse primer | *GCTACGACGTGGGCTACAG* |
| m-IFN-γ | Forward primer | *ATGAACGCTACACACTGCATC* |
|  | Reverse primer | *CCATCCTTTTGCCAGTTCCTC* |
| m-GZMB | Forward primer | *TGCAAGGAGGAAGTGTCAGG* |
|  | Reverse primer | *TGTAGTGGGCGGTGTCATAG* |

Table S3 The information on the antibodies used in this study

| Name | Resource | Lot number |
| --- | --- | --- |
| Rabbit monoclonal antibody against-HDAC2 | ABclonal (A22426) | 4700007005 |
| Rabbit polyclonal antibody against-NAT10 | Proteintech (13365-1-ap) | 00042575 |
| Rabbit polyclonal antibody against-ANKRD12 | Thermo (PA5-68605) | 35EF9A53 |
| Rabbit polyclonal antibody against-c-Myc | Proteintech (10828-1-ap) | 00138557 |
| Rabbit polyclonal antibody against-Histone H3ac (pan-acetyl) antibody | Proteintech (61637) | 3260200 |
| Mouse monoclonal antibody against-HA | ABclonal (AE008) | 9200008004 |
| HRP-conjugated Goat anti-Rabbit IgG (H+L) | ABclonal (AS014) | 3600011157 |
| HRP-conjugated Goat anti-Mouse IgG (H+L) | ABclonal (AS003) | 9300003001 |
| Anti β-Actin Mouse Monoclonal Antibody | CWBIO (CW0096M) | 01264/18723 |
| anti-ac4C | ABCAM (252215) | GR3269449-5 |

Table S4 Primer sequences for ChIP-qPCR

| Name | | Sequence (5’-3’) |
| --- | --- | --- |
| h-IFN-γ for ChIP | Forward primer | *AAATCAGAGTCCCGCGGTG* |
|  | Reverse primer | *CCCAGCAAAGATCCGGAAGT* |
| h-TNF-α for ChIP | Forward primer | *TGCTGATTAGAAGGCTCACTTGTG* |
|  | Reverse primer | *CCAGTAACCGTAATTCCCAGTCTTG* |
| h-GZMB for ChIP | Forward primer | *CACTTCATAGGCTTGGGTTCCTG* |
|  | Reverse primer | *CTCTGGGTGCTTGTGTGAGAATC* |
| h-KRAS for ChIP | Forward primer | *CTCCCAGCTCAGGACTTTGA* |
|  | Reverse primer | *GGAGGCTGAGGTAGTGTTGG* |
| h-CDK4 for ChIP | Forward primer | *GGGCGCTGGGAAGTAAAG* |
|  | Reverse primer | *CCCAGCTCCTCACTCCAAC* |

Table S5 Mutant site of circANKRD12 and c-Myc

| Gene | WT | MUT |
| --- | --- | --- |
| circANKRD12 | *CAACGCCTGCCC* | *GAAGGCGTGCCC* |
| c-Myc | *AAGAAATTCGAG* | *AAGAGATTCGAG* |

Table S6 Risk table corresponding to the survival curve in Figure 4K

| Overall Survival (days) | EV | circANKRD12-OE |
| --- | --- | --- |
| 0 | 10 | 10 |
| 20 | 9 | 6 |
| 40 | 5 | 1 |
| 60 | 4 | - |
| 80 | 2 | - |

Table S7 Risk table corresponding to the survival curve in Figure 7I

| Overall Survival (days) | EV | circANKRD12-OE |
| --- | --- | --- |
| 0 | 6 | 6 |
| 10 | 6 | 5 |
| 20 | 4 | 1 |
| 40 | 2 | - |
| 60 | 1 | - |

Supplementary Figure


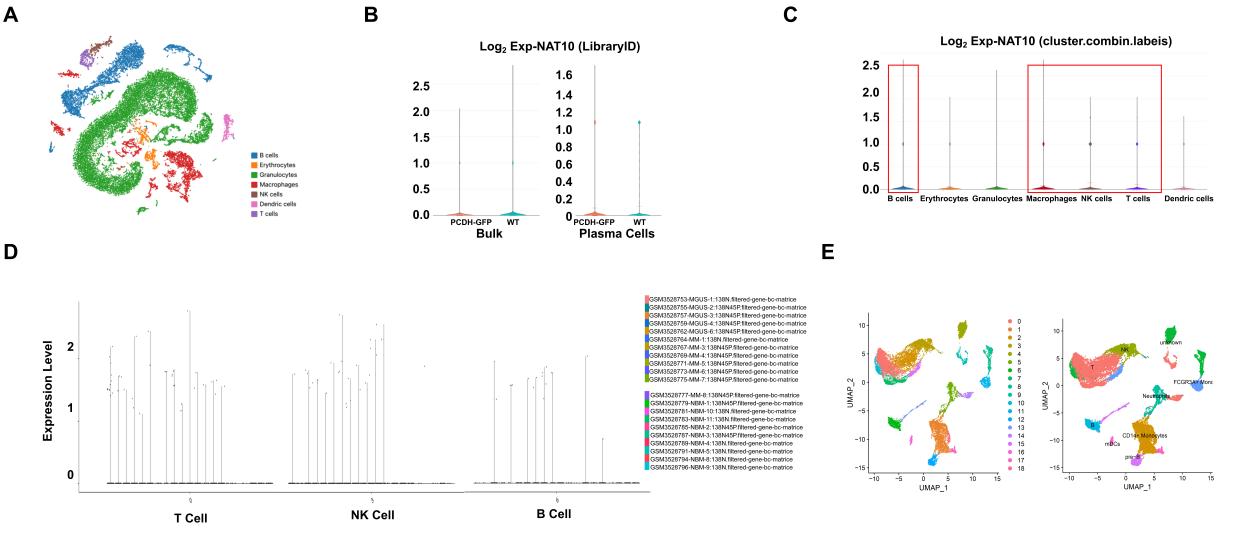
Fig. S1 NAT10 is highly expressed in immune cells of MM patients and mouse models.

A-C NAT10 expression was elevated in various immune cell types, including B cells, NK cells, T cells, and macrophages. D-E Reproducibility analysis of 10x Genomics single-cell sequencing data.


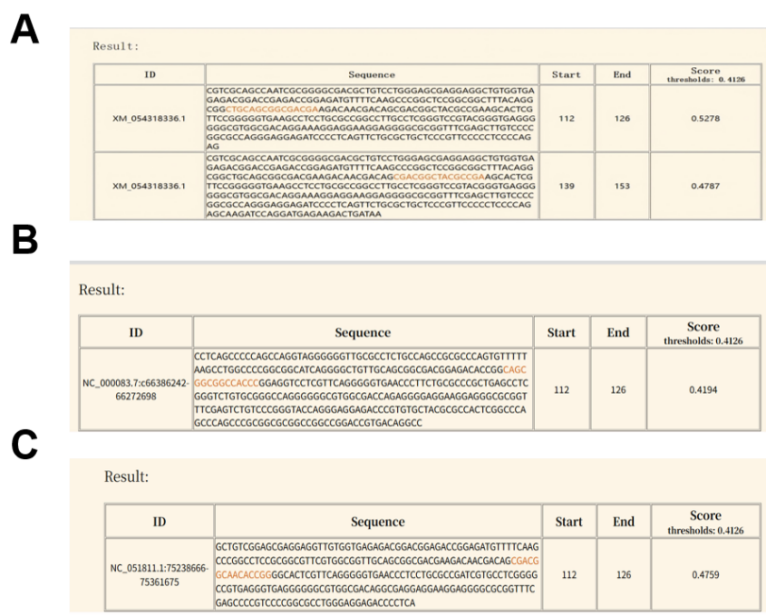


Fig. S2 Prediction of ANKRD12 acetylation sites in humans (A), mice (B), and dogs (C).
